# Prevalence, determinants, and wealth-related inequalities in malaria among under five children in Uganda: evidence from the malaria indicator survey

**DOI:** 10.1186/s40249-026-01479-w

**Published:** 2026-07-20

**Authors:** Edson Mwebesa, Delight Mawufemor Agbi, Isaac Isiko, Gideon Ikemdinachi Nwankwo, Rejoice Uche Obiora, Bisola Folusho Olubiyi, Charles Natuhamya

**Affiliations:** 1 Department of Mathematics, Faculty of Science, Muni University, Arua, Uganda; 2 Department of Mathematics, Computing and Physics, School of Science and Aerospace Studies, Moi University, Eldoret, Kenya; 3 Faculty of Medicine School of Population and Public Health, University of British Columbia, Vancouver, Canada; 4 Department of Health Management and Policy, Faculty of Health Sciences, American University of Beirut, Beirut, Lebanon; 5 College of Health, Lehigh University, Bethlehem, PA USA; 6 University of Global Health Equity, Butaro, Burera, Kigali, Rwanda; 7 Ugandan Youth Development Link, Kampala, Uganda

**Keywords:** Malaria, Under-five children, Socio-demographic, Wealth-related inequalities, Uganda

## Abstract

**Background:**

Malaria remains a leading cause of morbidity and mortality among children under five in Uganda. Despite national control efforts, significant disparities and inequalities in prevalence persist across regions, residences, and mean socio-economic status. This study examines the socio-demographic factors and wealth-related inequalities associated with malaria among children under five in Uganda.

**Methodology:**

A secondary analysis of data from the Uganda Malaria Indicator Survey (UMIS) 2018–2019 was conducted. A sample of 4,600 children with malaria test results was included in the study. The distribution of malaria prevalence across socio-demographic factors was analysed using cross-tabulations and a chi-squared test. Malaria-related inequalities were measured using equity plots and the concentration index (CIX). A multilevel logistic regression model was used to examine the relationship between malaria prevalence and associated factors. The results are presented as adjusted odds ratios (a*OR*s) with 95% confidence intervals (*CI*).

**Results:**

Almost twenty-three in every 100 children under five had malaria infection (95%* CI:* 19.2–27.0). Regional variations in malaria prevalence and wealth-related inequalities were observed. The multilevel model identified several significant independent factors: older child age (a*OR* = 2.12, 95% *CI:* 1.51–2.96, *P* < 0.001), child’s anemia (a*OR *= 3.16, 95% *CI:* 2.33–4.29, *P* < 0.001), and larger household size (a*OR* = 1.98, 95% *CI*: 1.13–3.45, *P* < 0.05) were positively associated with malaria in children under five years in Uganda. A negative concentration index (CIX = − 0.334, *P* < 0.001) was also observed, indicating that higher malaria prevalence is concentrated among children in the poorest wealth quintile.

**Conclusion:**

Malaria prevalence in Uganda is associated with a complex interplay of socioeconomic and geographic factors. The substantial disparities observed highlight the need for tailored public health strategies designed for high-burden regions and vulnerable communities to reduce disease burden effectively.

## Background

Malaria is among the most endemic and lethal infectious diseases in the world, and sub-Saharan Africa continues to contribute more than 90 percent of all malaria-related deaths, especially in children below the age of five years [1]. Malaria remains one of the main contributors to morbidity and mortality among these vulnerable groups in Uganda, despite decades of national and global work to reduce malaria transmission through vector control, enhancement of diagnostics, and access to more treatment modalities [2]. The disease not only poses threats to the survival of children; it is also a huge burden on families, the community, and the healthcare system.

The Malaria control strategy in Uganda has progressed to incorporate the extensive use of insecticide-treated nets (ITNs), indoor residual spraying (IRS), and the implementation of artemisinin-based combination therapies (ACTs) [2, 3]. These interventions have contributed to the gradual reduction in national malaria prevalence, but aggregate statistics tend to mask underlying differences in disease burden across geographic areas and socio-demographic groups. Evidence suggests that the prevalence of malaria is localised in certain regions and among vulnerable populations, which raises questions about the effectiveness of existing control measures [4, 5].

Malaria in children under five years of age is frequently more severe due to their underdeveloped immune systems. Environmental factors, such as high vector density and favourable climatic conditions conducive to mosquito breeding, in conjunction with social determinants, further enhance their susceptibility. These social determinants encompass maternal education level, household wealth, and access to malaria prevention measures [6, 7]. This situation poses an even greater risk to refugee populations, as they are particularly susceptible to displacement, overpopulation, and limited access to healthcare services [8, 9]. The overlapping susceptibilities lead to a situation in which the threat of malaria is not uniformly distributed but tends to be concentrated among the most disadvantaged individuals [10, 11].

Although malaria is preventable and treatable, its continued burden among children under five years in Uganda, particularly in high-burden areas and populations receiving limited or no programmatic attention, highlights gaps in intervention coverage and the limited effectiveness of existing control strategies. Moreover, nationally aggregated statistics often fail to capture the lived realities of children in remote rural communities, refugee settlements, and impoverished households, where access to health services and preventive measures remains severely constrained [1, 2]. In the absence of granular knowledge about the socio-demographic determinants and wealth-related inequalities of malaria, the fight against the disease risks leaving children and, in general, individuals who need it the most [12, 13].

This study aims to bridge this gap by identifying inequalities in the prevalence of malaria among children under five years old in Uganda based on data collected in the 2018–2019 Uganda Malaria Indicator Survey (UMIS). The UMIS provides nationally representative data on a wide range of health indicators, including malaria, assessed by rapid diagnostic tests and microscopy [2]. Through a multilevel logistic regression, the study considers the hierarchical level of the data, children within households and communities, and examines the independent associations with malaria at four levels: child, maternal, household, and community [5, 14, 15]. Equity plots and the concentration index were used to assess the magnitude of wealth-related inequalities in malaria prevalence across geographic regions and places of residence. This study, therefore, aimed to examine disparities and wealth-related inequalities in malaria prevalence and to identify socio-demographic factors associated with malaria prevalence among children under five in Uganda. This is helpful to Uganda in lowering child mortality and moving towards the Sustainable Development Goal (SDG) 3: Ensuring healthy lives and promoting well-being at all ages [1, 16, 17].

## Methods

### Study design and data source

This study employed a quantitative, cross-sectional design, utilising secondary data from the 2018–2019 UMIS [2]. The MIS is a nationally representative household survey designed to generate precise and reliable estimates of malaria prevalence and related health indicators across Uganda. The UMIS was conducted in 15 sub-regions of Uganda and 9 refugee settlements, with 8,351 households selected. A two-stage sample design was used, in which clusters were selected in the first stage, followed by the selection of households with children under 5 years [2]. In this study, Household Recode (HR) and Child Recode (CR) were merged because the former included variables on malaria prevalence among children, and the latter included child-, woman (mother)-, and context-level variables. After merging, the sample comprised 4,600 children with malaria test results. This is the sample that was used in this study.

### Study variables

The primary outcome variable was malaria status, defined as a binary variable (positive vs negative) based on malaria test results in UMIS. Explanatory variables were grouped into four categories: child-level factors [sex of the child (male, female), age of the child (< 1 year, 1–2 years, 3 or more years), child slept under an insecticide-treated net (yes, no), child anemia (yes, no), maternal-level factors (age of the mother, education level of the mother, number of children ever born), household-level factors (sex of household head, number of household members, wealth index) and community-level factors (place of residence and region). This classification enabled a comprehensive, multi-layered assessment of malaria risk on malaria infection in Uganda.

### Statistical analysis

All analyses were performed in Stata version 17.0 [18]. Survey weights, clustering, and stratification were accounted for to adjust for the complex sampling design. Descriptive statistics were used to summarise the characteristics of the study sample and to estimate malaria prevalence. Categorical variables were summarised using percentages and their 95% confidence intervals (*CI*). Cross-tabulations of malaria prevalence by socio-demographic factors (row percentages) were done to identify the disparities in malaria prevalence, and associations between malaria prevalence and socio-demographic factors were tested using a chi-square test. To assess the factors associated with malaria prevalence while accounting for the hierarchical nature of the data (children nested within clusters or enumeration areas), a binary logistic mixed-effects regression model was fitted. Due to this clustering, traditional logistic regression models, which assume independence among observations, would fail to account for cluster-level variation. Mixed-effects logistic models account for such group- or cluster-level variations, allow the simultaneous assessment of individual- and group-level factors, and offer better estimates [19, 20].

The general form of the model was:

$$\log \left( {\frac{{p_{ij} }}{{1 - p_{ij} }}} \right) = \beta_{0} + \beta_{1} X_{1ij} + \cdots + \beta_{k} X_{kij} + u_{j}$$

where ${p}_{ij}$ is the probability of malaria for the child *i* within cluster *j*, β0 is a fixed intercept, β1,…, β*k* are fixed-effect coefficients for explanatory variables, ${X}_{1ij},\dots , {X}_{kij}$. ${u}_{j}$ is the random effect for the cluster $j$, which is the deviation of the cluster’s intercept from the overall fixed effect and ${u}_{j} \sim N(0, {\sigma}_{u}^{2})$.

Four nested models were fitted to examine the incremental effect of adding covariates: Model 1 (Null Model): Included only the random effect for clusters to estimate the baseline Intraclass Correlation Coefficient (ICC) and check whether there is a need for multilevel modelling or if ICC was high. An ICC less than 10% would indicate no need for multilevel modelling, while a higher ICC would suggest variation between clusters. Model 2: Added child-level factors, Model 3: Added maternal, household, and community-level factors, and Model 4 (Final Model): Included variables from previous models whose *P*-values were less than or equal to 20% to estimate independent effects after adjusting for all covariates. These variables were added in the model to ensure that potential factors are not excluded prematurely and that variables that may become significant after adjustment for other factors are not excluded. In addition, key socioeconomic and demographic variables (including wealth index, maternal education, household size, place of residence, and region) were retained in the final model based on their established role as confounders in the malaria literature, regardless of statistical significance, to ensure adequate control for confounding. Results were reported as adjusted odds ratios (a*OR*s) with 95% *CI*s. The Akaike Information Criterion (AIC) was used to assess model fit, with lower values indicating a better-fitting model. A *P*-value of < 0.05 was considered statistically significant. The ICC and cluster variations were also presented with their 95% *CI*s. Multicollinearity among factors was assessed using the Variance Inflation Factor (VIF), with a threshold of VIF > 10 indicating multicollinearity. All variables had VIF values less than 5 (maximum VIF = 3.0), with a mean VIF of 1.80. This indicated acceptable levels of multicollinearity. Tolerance (1/VIF) values were all above 0.3, further confirming that multicollinearity was not a big problem. These findings showed that the included factors were sufficiently independent to produce stable regression estimates.

### Measuring wealth-related inequality

To measure wealth-related inequalities in malaria prevalence among children under 5 in Uganda, this study used equity plots and the concentration index. Equity plots were used to visualise absolute differences in malaria prevalence across wealth quintiles within sub-regions and places of residence. Malaria prevalence was a binary outcome (1 = positive, 0 = negative). For each subgroup, weighted prevalence estimates were calculated using the survey sampling weights provided in the MIS data. Prevalence was obtained using weighted means and expressed as percentages. The variable *wealth index combined* (v190) in the MIS data, categorised as poorest, poorer, middle, richer, and richest, was used. For analyses within sub-regions, prevalence estimates were obtained separately for each region-wealth quintile combination, preserving the national wealth ranking. Equity plots allow assessment of the absolute gap between the poorest and richest quintiles within each sub-region and each place of residence.

The magnitude of wealth-related inequality was obtained using the concentration index (CIX), which measures the degree to which malaria prevalence is concentrated across the socioeconomic distribution. The index was computed as:

$$CIX = \frac{2}{\mu }COV\left( {y_{i} , r_{i} } \right)$$

where ${y}_{i}$ is malaria outcome in child $i$, $\mu$ is the mean prevalence, ${r}_{i}$ is the fractional rank of children in the wealth distribution based on wealth index (v191), which is a continuous wealth variable in MIS data. Since malaria status among children is a binary outcome, the standard CIX was computed with the “truezero” option to account for the bounded nature of the variable. Concentration curves were generated to complement the CIX. This plotted the cumulative proportion of malaria cases against the cumulative proportion of the children ranked by wealth.

**Fig. 1:**
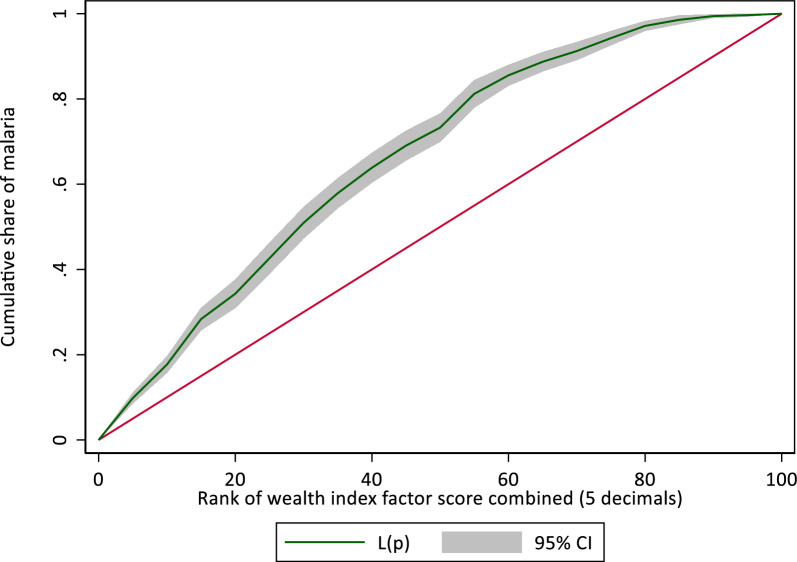
Concentration Curve. The curve plots the cumulative proportion of malaria cases (*y*-axis) against the cumulative proportion of children ranked from poorest to richest (*x*-axis). The diagonal line represents perfect equality. A curve lying below the diagonal indicates that malaria is disproportionately concentrated among poorer children (pro-poor inequality), consistent with the negative concentration index (CIX =  − 0.334, *P* < 0.001) observed in this study

In the equity plots, the *x*-axis represents wealth quintiles ordered from poorest (left) to richest (right), and the* y*-axis represents malaria prevalence (%). A downward slope from left to right indicates pro-poor inequality, where poorer children have higher malaria prevalence. The absolute gap between the poorest and richest quintile bars represents the wealth-related equity gap within each sub-region or place of residence. In the concentration curve (Fig. 1), the *x*-axis shows the cumulative proportion of children ranked from poorest to richest, and the *y*-axis shows the cumulative proportion of malaria cases. A curve lying below the diagonal line of perfect equality indicates that malaria is disproportionately concentrated among the poor. Based on CIX, a negative value indicates a pro-poor concentration of disease, a value of zero reflects perfect equality across the wealth distribution, and a positive value indicates that malaria is more concentrated among wealthier groups. The further the CIX is from zero, the greater the degree of wealth-related inequality.

## Results

### Disparities in malaria prevalence by socio-demographic characteristics

The results reveal that the overall prevalence of malaria among children under five was 22.9% (95% *CI:* 19.2–27.0%). The results further reveal that malaria prevalence increased with children’s age, from 20.0% (95%* CI:* 16.0–24.7%) among children younger than 1 year to 25.0% (95%* CI:* 20.9–29.6%) among those aged 3 or older (*P* = 0.023). Children who did not sleep under a mosquito net had a higher prevalence of 30.2% (95% *CI:* 23.4–38.0%) than those who did (21.2%, 95% *CI:* 17.8–25.1%, *P* = 0.003). The highest prevalence was observed among children with anaemia, at 32.1% (95% *CI:* 26.4–38.5%), compared with non-anaemic children, at 15.0% (95%* CI:* 12.2–18.3%; *P* < 0.001). Among maternal factors, the results reveal that the prevalence of malaria reduced with increasing level of maternal education, with 38.9% (95%* CI:* 31.3–47.1%) among those with no education to 8.3% (95%* CI:* 3.4%–18.8%) among women with higher education level (*P* < 0.001). On the other hand, the results showed that the prevalence of malaria among children under five increased with increasing number of children ever born, with 16.7% (95%* CI:* 12.9–21.3%) for women who gave birth 1 to 2 children and 28.1% (95%* CI:* 22.7–34.2%) among those who gave birth to five or more children (*P* < 0.001). Household factors were also associated with disparities in malaria prevalence. Children from female-headed households were associated with a higher prevalence, 28.2% (95% *CI:* 22.5–34.7%), than those from male-headed households, 20.9% (95% *CI:* 17.2–25.1%, *P* = 0.011). Larger households had a greater burden, with prevalence rising from 11.9% (95%* CI:* 8.4–16.7%) in households with 1–3 members to 27.9% (95% *CI:* 22.8–33.5%) in households with 7 or more members (*P* < 0.001). The study also observed that the poorest households had a higher prevalence of 39.0% (95% *CI:* 31.8–46.7%), compared with the richest households at 2.6% (95% *CI:* 1.4–4.9%, *P* < 0.001). In terms of place of residence, refugee children had the highest prevalence of malaria, 41.8% (95% *CI:* 28.9–55.8%), followed by children residing in rural areas, 25.6% (95% *CI: *21.0–30.9%), while children residing in urban areas had the lowest prevalence, 6.6% (95% *CI:* 3.5–12.2%, *P* < 0.001). Results are shown in Table 1.

**Table 1:** Prevalence of malaria among children under five years in Uganda by socio-demographic and community-level characteristics

	Overall	Malaria positive	*P*-value
	% (95%* CI*)	% (95% *CI*), row	
Child-level factors			
Sex of child			
Male	50.8 (49.1–52.6)	22.9 (19.3–26.9)	0.997
Female	49.2 (47.4–50.9)	22.9 (18.6–27.7)	
Current age of children			
< 1 year	21.0 (19.4–22.7)	20.0 (16.0–24.7)	0.023
1–2 years	38.9 (36.9–41.0)	22.7 (18.7–27.3)	
3 or more years	40.1 (38.5–41.7)	25.0 (20.9–29.6)	
The child lives with whom			
Respondent	96.1 (95.0–97.0)	23.4(19.6–27.6)	0.059
Lives elsewhere	3.9 (3.0–5.0)	14.8 (8.7–24.1)	
The child slept under a mosquito net			
None	25.5 (23.0–28.1)	30.2 (23.4–38.0)	0.003
Yes (All/some children)	74.6 (71.9–77.1)	21.2 (17.8–25.1)	
Child’s anaemia			
No	48.9 (46.0–51.8)	15.0 (12.2–18.3)	< 0.001
Yes	51.1 (48.2–54.0)	32.1 (26.4–38.5)	
Mother-level factors			
Age of the mother			
15–24	26.6 (23.8–29.6)	21.3 (16.7–26.7)	0.549
25–29	26.0 (23.4–28.8)	24.8 (19.7–30.7)	
30–49	47.4 (43.1–51.8)	22.7 (18.3–27.9)	
Maternal level of education			
No education	19.3 (16.7–22.2)	38.9 (31.3–47.1)	< 0.001
Primary	53.8 (50.3–57.3)	24.3 (20.1–29.2)	
Secondary	22.4 (19.6–25.5)	8.5 (5.9–12.0)	
Higher	4.5 (3.2–6.3)	8.3 (3.4–18.8)	
Number of children ever born			
1–2 children	25.8 (22.9–28.9)	16.7 (12.9–21.3)	< 0.001
3–4 children	30.2 (28.1–32.3)	20.5 (16.5–25.1)	
5 or more	44.1 (40.1–48.1)	28.1 (22.7–34.2)	
Household factors			
Sex of household head			
Male	72.5 (69.7–75.3)	20.9 (17.2–25.1)	0.011
Female	27.5 (24.8–30.3)	28.2 (22.5–34.7)	
Number of household members			
1–3 members	13.1 (11.2–15.1)	11.9 (8.4–16.7)	< 0.001
4–6 members	48.7 (46.5–50.9)	21.9 (18.1–26.2)	
7 or more members	38.3 (35.7–40.9)	27.9 (22.8–33.5)	
Wealth index			
Poorest	29.0 (25.4–32.8)	39.0 (31.8–46.7)	< 0.001
Poorer	20.7 (18.2–23.4)	26.5 (20.6–33.4)	
Middle	16.7 (13.9–19.9)	22.5 (17.0–29.2)	
Richer	15.7 (13.4–18.3)	12.0 (8.1–17.3)	
Richest	18.0 (12.8–24.7)	2.6 (1.4–4.9)	
Community-level factors			
Type of place of residence			
Urban	23.6 (17.5–31.1)	6.6 (3.5–12.2)	< 0.001
Rural	65.6 (58.4–72.2)	25.6 (21.0–30.9)	
Refugee	10.7 (8.0–14.2)	41.8 (28.9–55.8)	
Region			
Kampala	3.2 (2.3–4.4)	2.0 (0.4–9.4)	< 0.001
South Buganda	14.6 (10.3–20.5)	3.5 (1.0–5.9)	
North Buganda	14.8 (10.1–21.2)	19.3 (8.9–36.9)	
Busoga	7.9 (6.3–9.9)	53.6 (37.3–69.2)	
Bukedi	4.4 (3.5–5.6)	11.2 (3.9–28.0)	
Bugisu	5.5 (3.7–8.0)	12.1 (3.8–32.1)	
Teso	4.6 (3.6–5.8)	28.8 (16.5–45.3)	
Karamoja	4.0 (3.1–5.2)	45.1 (32.4–58.4)	
Lango	5.6 (3.4–6.1)	24.1 (12.1–42.2)	
Acholi	3.7 (2.5–5.5)	42.7 (26.6–60.6)	
West Nile	10.3 (7.7–13.6)	51.5 (42.8–60.0)	
Bunyoro	4.1 (3.0–5.5)	13.2 (7.3–22.8)	
Tooro	8.5 (5.6–12.6)	28.0 (16.4–43.5)	
Ankole	6.4 (4.3–9.4)	2.4 (0.3–16.1)	
Kigezi	3.4 (2.8–4.2)	0 (0–0)	

The prevalence of malaria among under-five children was 22.9% (95%* CI:* 19.2–27.0%)

### Disparities in malaria prevalence by region

The results further show variation in malaria prevalence among children under five across Uganda’s sub-regions. A significantly lower rate in the central and southwestern parts of the country and a high burden concentrated in the northern, northeastern, and northwestern regions were observed. The lowest prevalence is observed in Kigezi (0%), Kampala (2%), and Ankole (2.4%), indicating minimal malaria transmission in these areas. Other sub-regions with relatively low rates include South Buganda (3.5%), Bukedi (11.2%), Bugisu (12.1%), and Bunyoro (13.2%). In contrast, the highest prevalence is recorded in Busoga (53.6%), West Nile (51.5%), and Karamoja (45.1%), followed by Acholi (42.7%). These sub-regions are likely influenced by environmental, ecological and socioeconomic conditions that favour malaria transmission, such as high vector density and climatic factors. Mid-range prevalence is seen in Teso (28.8%), Lango (24.1%), and Tooro (28.0%). Results are shown in Table 1.

### Socio-demographic factors associated with the prevalence of malaria among children under five in Uganda

The results from multilevel logistic regression revealed that the likelihood of malaria among children aged 1–2 years (a*OR* = 1.42, 95% *CI:* 1.04–1.93) and 3 or more years (a*OR* = 2.12, 95%* CI:* 1.51–2.96) was higher compared to those aged less than one year. Children aged 3 or more years were twice as likely to have malaria compared to those who were less than one-year-old. The results further revealed that a child with anaemia was more than three times more likely to have malaria (aOR = 3.16, 95% *CI* 2.33–4.29) compared to those without anaemia. A mother having a primary level of education was negatively associated with malaria among children (a*OR* = 0.45, 95%* CI:* 0.24–0.85) compared to no education. This implies that children whose mothers had a primary level of education were 55% less likely to have malaria compared to those with no education. Having more household members in a household was positively associated with malaria among children under five years (a*OR* = 1.80, 95% *CI:* 1.05–3.08) for households with 4–6 members and (a*OR *= 1.98, 95% *CI:* 1.13–3.45) for households with 7 or more members compared to those with one to three members. This shows that an increasing number of household members is associated with high odds of malaria among children under five years. The results revealed that malaria prevalence decreased with increasing household wealth index, with (a*OR *= 0.37, 95% *CI:* 0.18–0.75) for richer households and (a*OR* = 0.13, 95% *CI:* 0.05–0.33) for the richest households compared to the poorest households. Children from rural areas were more likely to have malaria (a*OR* = 3.57, 95%* CI: *1.55–8.20) than those from urban areas. Similarly, children from refugee settlements were almost 11 times more likely to have malaria (a*OR* = 10.95, 95% *CI:* 3.45–34.74) compared to those from urban residences. This highlights a disproportionately high malaria prevalence among refugees compared to other children. The ICC decreased from 0.70 in the empty model to 0.63 in the final model, indicating that the included covariates explained some of the between-cluster variation and supporting the appropriateness of a multilevel modelling approach. All associations reported are from a cross-sectional survey and should be interpreted as statistical associations rather than causal relationships. Results are shown in Table 2.

**Table 2:** Multilevel mixed-effects logistic regression models examining disparities in malaria prevalence

Covariates	Model 1	Model 2	Model 3	Model 4
		a*OR* (95%* CI*)	a*OR* (95% *CI*)	a*OR* (95%* CI*)
Child level factors				
Current age of children				
< 1 Year	–	Reference	–	Reference
1–2 years	–	1.36 (1.01–1.83)*	–	1.42 (1.04–1.93)*
3 or more years	–	2.17 (1.57–3.01)***	–	2.12 (1.51–2.96)***
The child slept under a mosquito net				
None	–	Reference	–	Reference
Yes (All/some)	–	0.68 (0.44–1.06)^	–	0.73 (0.48–1.11)
Child's anaemia				
No	–	Reference	–	Reference
Yes	–	3.30 (2.44–4.46)***	–	3.16 (2.33–4.29)***
Mother-level factors				
Maternal level of education				
No education	–	–	Reference	Reference
Primary	–	–	0.94 (0.63–1.41)	0.93 (0.61–1.42)
Secondary	–	–	0.44 (0.212–0.87)*	0.45 (0.24–0.85)*
Higher	–	–	0.81 (0.28–2.29)	0.84 (0.28–2.52)
Number of children ever born				
1–2 children	–	–	Reference	–
3–4 children	–	–	1.15 (0.74–1.79)	–
5 or more	–	–	1.05 (0.69–1.59)	–
Household factors				
Sex of household head				
Male	–	–	Reference	–
Female	–	–	0.95 (0.67–1.34)	–
Number of household members				
1–3 members	–	–	Reference	Reference
4–6 members	–	–	2.53 (1.53–4.17)***	1.80 (1.05–3.08)*
7 or more members	–	–	2.82 (1.55–5.15)***	1.98 (1.13–3.45)*
Wealth index				
Poorest	–	–	Reference	Reference
Poorer	–	–	0.86 (0.53–1.39)	0.94 (0.58–1.52)
Middle	–	–	0.75 (0.43–1.31)	0.71 (0.40–1.27)
Richer	–	–	0.38 (0.18–0.79)**	0.37 (0.18–0.75)**
Richest	–	–	0.11 (0.04–0.28)***	0.13 (0.05–0.33)***
Community-level factors				
Type of place of residence				
Urban	–	–	Reference	Reference
Rural	–	–	3.68 (1.67–8.11)***	3.57 (1.55–8.20)**
Refugee	–	–	9.90 (3.31–29.68)***	10.95 (3.45–34.74)***
Random effect				
PSU variance (95%*CI*)	7.70 (5.80–10.21)	7.74 (5.77–10.39)	5.63 (4.10–7.73)	5.72 (4.15–7.89)
ICC (95% *CI*)	0.70 (0.64–0.76)	0.70 (0.64–0.76)	0.63 (0.55–0.70)	0.63 (0.56–0.71)
Model fit statistics				
AIC	3249.4	2847.6	3107.8	2745.6
Number of clusters	339	338	339	338

^***^ *P* < 0.001, **P < 0.01, *P < 0.05, ^*P* < 0.20

Model 1 is the null model, with only the outcome. This was used to evaluate whether a mixed-effects model is needed, based on the intraclass correlation coefficient (ICC). Model 2 has only the child-level factors, Model 3 combines the mother-level, household-level and community-level factors. Model 4 combines all factors from Models 2 and 3 with *P*-value < 0.20 to form the final model. Where the sign”–” is used, it implies that that particular factor was not included in the model. *PSU* Primary sampling unit, *AIC* Akaike Information Criterion

### Wealth-related inequalities in malaria prevalence among under-five children in Uganda

The findings of this study highlight a significant social gradient in malaria prevalence among children under five in Uganda, as evidenced by both the visual equity plots and the CIX (CIX = − 0.334, *P* < 0.001). Nationally, the negative CIX value confirms a statistically significant pro-poor distribution of malaria prevalence, indicating that the burden is disproportionately concentrated among children from the lowest wealth quintiles. These results are visually displayed in Fig. 1.

**Fig. 2:**
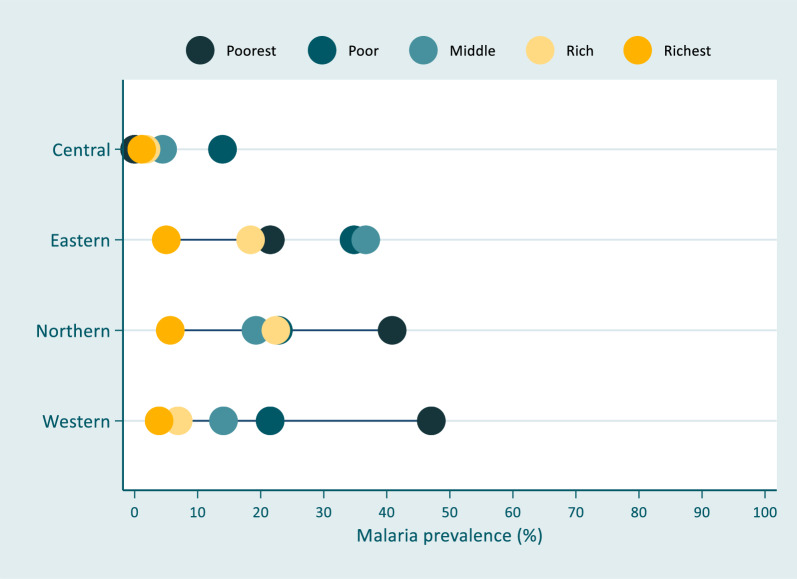
Equity plot of malaria prevalence by regions (recoded) in Uganda. Each bar represents the absolute malaria prevalence for a given wealth quintile within a region. The gap between the leftmost bar (poorest) and the rightmost bar (richest) represents the absolute wealth-related equity gap in that region. Wider gaps indicate greater inequality. Regions are sorted by overall prevalence to facilitate comparison

Regional disaggregation through equity plots shows both geographical and socioeconomic inequality. The Central region demonstrated relatively low prevalence and narrow wealth gaps. In contrast, the Western and Northern regions exhibited expansive equity tails, where the poorest children faced a malaria risk up to four times higher than their wealthiest peers. Results are shown in Fig. 2.

**Fig. 3:**
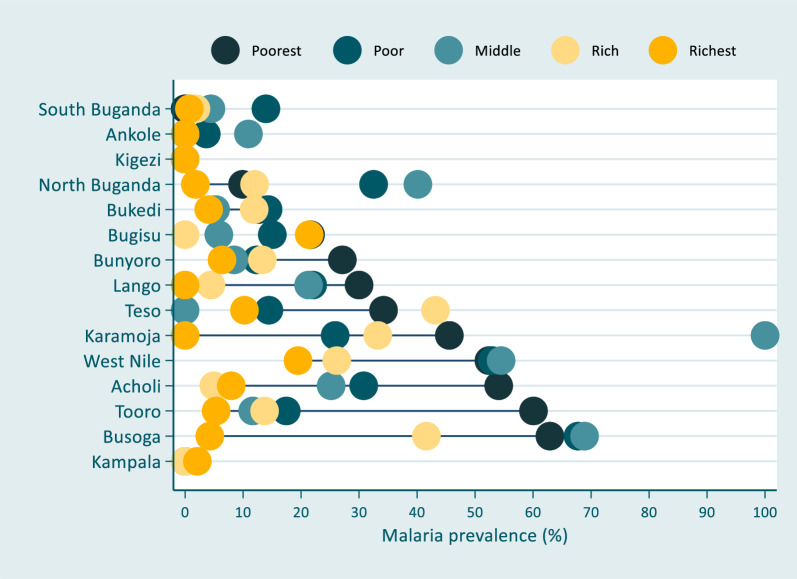
Equity plot of malaria prevalence by regions in Uganda. Equity plots for all 15 sub-regions in Uganda show malaria prevalence (*y*-axis) by wealth quintile (*x*-axis, poorest to richest). Each line represents one sub-region. A steep downward slope from left to right indicates a strong wealth gradient, with poorer children bearing a much higher malaria burden than wealthier peers in the same sub-region

In Fig. 3, the study presented all 15 sub-regions in Uganda. The results from the equity plot show that, nationally, the prevalence of malaria is disproportionately borne by the poorest quintiles. Sub-regional disaggregation reveals that while urbanised or high-altitude areas like Kampala and Kigezi have achieved low, equitable prevalence, sub-regions such as Busoga, Tooro, Karamoja and Acholi are characterised by massive equity tails. In these areas, the wealthiest children are effectively protected (prevalence < 10%), whereas their poorest counterparts remain highly vulnerable, with prevalence exceeding 50% in Acholi, Tooro, and Busoga, respectively. This suggests that current malaria control interventions are not reaching the most economically disadvantaged populations with the same efficacy as wealthier groups.

**Fig. 4:**
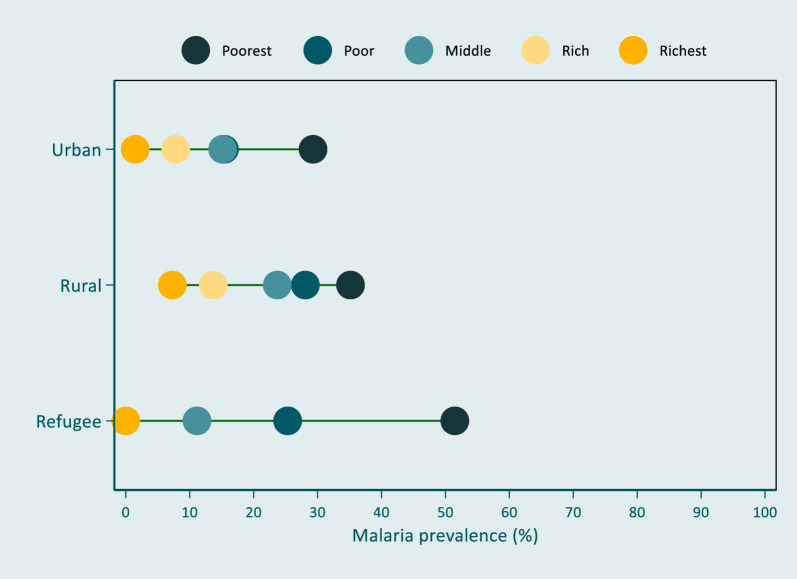
Equity plot of malaria prevalence by place of residence in Uganda. Equity plots disaggregated by place of residence (urban, rural, and refugee settlements) show malaria prevalence (*y*-axis) by wealth quintile (*x*-axis). Each curve represents one residential setting. The horizontal distance between the poorest and richest quintiles indicates the absolute equity gap in that setting. Refugee settlements show the widest equity gap, reflecting the greatest wealth-related inequality in malaria prevalence

Disaggregated analysis by type of place of residence reveals that the wealth-related equity gap in malaria prevalence is most pronounced among refugee children, where the absolute difference between the poorest and richest children reaches approximately 50 percentage points. While Urban areas maintained the lowest overall prevalence across all wealth quintiles, Rural settings displayed a higher baseline risk, with even the wealthiest children facing higher prevalence rates than their urban counterparts. These findings suggest that household wealth is moderated by the living environment, necessitating highly targeted, pro-poor interventions in refugee and rural communities to bridge significant health disparities. Results are shown in Fig. 4.

## Discussion

The results of this study indicate that malaria prevalence among Ugandan children under five remains substantial and unequally distributed across socio-demographic groups. The overall prevalence of 22.9% is lower than the 27.4% reported in a multi-country study across 13 sub-Saharan African countries [21], likely reflecting differences in transmission intensity, intervention coverage, and contextual factors. A clear age gradient was observed, with children aged three years and older significantly more likely to be infected than infants. These findings may be explained by the gradual waning of maternally acquired immunity during early childhood, coupled with increased mobility and exposure to mosquito bites as children grow older [22]. Similar age-related increases in malaria risk have been reported in studies across Africa. [9]. In Uganda, the lower use of insecticide-treated nets among older children than among younger children may further contribute to this elevated risk [7].

Substantial geographic disparities in malaria prevalence were observed across Uganda. Children residing in Busoga, West Nile, Karamoja, and Acholi experienced markedly higher prevalence than those living in Kampala and other urban areas. This pattern is consistent with growing evidence that malaria transmission in Africa is increasingly heterogeneous, with rural and disadvantaged areas bearing a disproportionate burden [19, 22]. Higher transmission in these regions may reflect a combination of environmental conditions favourable to mosquito breeding, limited access to healthcare services, lower coverage of effective malaria interventions, and weaker health-system capacity [23]. In refugee-hosting areas such as West Nile, population displacement and dependence on humanitarian services may further constrain access to prevention and treatment measures, increasing vulnerability to infection.

The strong association observed between malaria and anaemia in this study is both biologically plausible and consistent with previous evidence. Repeated *Plasmodium falciparum* infections contribute to red blood cell destruction, impaired erythropoiesis, and altered iron metabolism, thereby increasing the risk of anaemia among young children [24]. The coexistence of these conditions represents an important public health concern because both contribute substantially to childhood morbidity and mortality. Although the cross-sectional nature of the study limits causal inference, the findings support existing evidence from sub-Saharan Africa that improvements in malaria control are often accompanied by reductions in childhood anaemia and related adverse health outcomes [25].

This study also highlights the importance of socioeconomic and household factors in shaping malaria risk. Children whose mothers had no formal education were more likely to be infected, a finding that aligns with studies from the Democratic Republic of Congo and other African countries [26, 27]. Maternal education is often associated with improved health literacy, greater uptake of preventive measures, and more timely healthcare seeking. Similarly, malaria prevalence decreased with increasing household wealth, suggesting that economic resources provide protection through improved housing conditions, reduced overcrowding, and greater access to preventive tools such as insecticide-treated nets and indoor residual spraying [11]. Household size was also associated with malaria prevalence, with children living in larger households facing a higher risk. This may reflect increased crowding, limited availability of preventive resources, and competing household demands, all of which affect healthcare utilisation and disease prevention practices [11].

A major contribution of this study is the identification of significant pro-poor inequality in malaria prevalence among children under five in Uganda. The negative concentration index indicates that malaria disproportionately affects children from poorer households, consistent with evidence from Ghana and other sub-Saharan African settings [14, 28]. The inequality was particularly pronounced in several sub-regions and among refugee populations, where the burden of malaria remains high despite ongoing control efforts. While some regions exhibited clear wealth gradients in malaria prevalence, others, such as Karamoja, experienced persistently high prevalence across all wealth groups, suggesting that ecological and contextual factors may limit the protective effect of socioeconomic advantage. These findings indicate that universal malaria control strategies may not be benefiting all population groups equally. Achieving Uganda's malaria reduction and elimination targets will therefore require equity-focused approaches that prioritise poor households, high-burden regions, and refugee communities, while addressing the structural barriers that continue to drive malaria transmission among the most vulnerable populations.

### Strengths and limitations of the study

This study utilised nationally representative Uganda MIS data collected in 2018–2019. These nationally representative findings provide useful information for decision-making on malaria prevention interventions and resource allocation for children under five. In addition, the study shows the distribution of malaria among children across regions and other characteristics, providing further information for targeted interventions. The study utilised a Mixed-Effects modelling approach, which appropriately accounts for variation across clusters and thus better handles dependencies within groups, leading to more reliable estimates than traditional binary logistic regression models. The study highlights different factors that are associated with malaria among children under five in Uganda. The study also investigated wealth-related inequalities in malaria among children under five in Uganda, providing evidence to inform targeted interventions. The study analysed data collected from the 2018/19 malaria indicator survey, the most current MIS available in Uganda. The distributions of malaria may have changed since this survey was conducted. However, as this is a population-based dataset, the study’s validity remains high, with the potential to inform Uganda’s efforts to design strategies for controlling, preventing, and managing malaria among children under five years. This study found that child anaemia status was associated with malaria among children in Uganda. This relationship is complex and multifaceted, as malaria is known to be directly associated with anaemia among infected children.

On the other hand, there is also evidence of an inverse relationship between anaemia (such as iron-deficiency anaemia) and malaria. In this study, however, we observed a positive relationship between a child’s anaemia and malaria infection. Future studies would investigate further this to shed more light on such an outcome. Future studies could also use qualitative data to gain an in-depth understanding of contextual factors influencing malaria distribution and prevalence in Uganda. Unmeasured factors, such as those related to cultural values, norms, and attitudes towards malaria prevention uptake, may be associated with malaria infection among children but are not included in this study. Additionally, several important limitations warrant acknowledgement. First, malaria diagnosis in the UMIS relied primarily on rapid diagnostic tests (RDTs). While RDTs are the standard tool for population-based surveys, they are known to have imperfect sensitivity and specificity, particularly for detecting low-density parasitaemia. False-positive results may occur due to persistent HRP2 antigenemia following recent infections, and false negatives may arise in cases of low parasite density. These measurement errors could lead to some misclassification of malaria status, potentially biasing prevalence estimates and associations. Second, despite the comprehensive set of covariates included in the multilevel models, residual confounding remains possible.

The intraclass correlation coefficient (ICC) decreased slightly after adjusting for covariates, indicating that the included variables explained a small proportion of the between-cluster variation in malaria prevalence among children under five in Uganda. The persistently high ICC values across all models (ranging from 0.63 to 0.70) indicate that a substantial share of malaria risk is attributable to unobserved community-level factors, even after adjusting for all included covariates. This highlights the importance of contextual factors on malaria transmission, such as ecological conditions, such as rainfall, altitude, and temperature; environmental factors, such as proximity to mosquito breeding sites, which are not usually captured in MIS data. Future studies integrating ecological and spatial data to explain better residual clustering are imperative. These findings emphasise that malaria control strategies should incorporate geographically targeted interventions that address local transmission dynamics, rather than relying solely on individual-level risk factors. Third, the data used in this study were collected in 2018–2019, which limits the contemporaneous policy relevance of the findings given subsequent changes in Uganda’s malaria burden, intervention coverage, and disease landscape. Nonetheless, the 2018–2019 UMIS remains the most recent nationally representative malaria indicator survey for Uganda, and the structural determinants identified are unlikely to have fundamentally changed, making the findings still informative for programmatic decision-making.

## Conclusion

Malaria among children under five in Uganda remains both prevalent and unequally distributed. Prevalence is associated not only with factors such as age and anaemia, but also with social determinants and household conditions, including poverty, crowding, maternal education, and geography. Children in rural and refugee settings bear the heaviest burden of malaria, and addressing these disparities requires targeted interventions that combine contextual and structural approaches. In high-prevalence regions such as Busoga, West Nile, Karamoja, and Acholi, Uganda should focus on expanding access to long-lasting insecticide-treated nets that are matched to local resistance patterns. The existence of wealth-related inequalities in malaria prevalence indicates that interventions that target poor communities and households are pivotal if malaria elimination goals are to be achieved. Also, strengthening anaemia prevention and post-discharge care for young children, and extending surveillance and outreach to hard-to-reach rural and refugee communities, are crucial steps. In addition, improving housing with affordable measures such as screening, closing eaves, and using better construction materials can complement biomedical efforts and contribute to sustained malaria reduction.

## Data Availability

The datasets analysed during the current study were obtained from the MEASURE DHS program. Data can be obtained at: https://dhsprogram.com/data/dataset_admin/index.cfm.

## Abbreviations

AIC: Akaike information criterion
a*OR*: Adjusted odds ratio
*CI*: Confidence interval
CIX: Concentration index
CR: Child recode
HR: Household recode
ICC: Inter-cluster correlation coefficient
RDTs: Rapid diagnostic tests
UMIS: Uganda malaria indicator survey
